# Optimizing FPGA implementation of high-precision chaotic systems for improved performance

**DOI:** 10.1371/journal.pone.0299021

**Published:** 2024-04-09

**Authors:** Issam Damaj, Ashraf Zaher, Wafic Lawand

**Affiliations:** 1 Department of Engineering, Cardiff School of Technologies, Cardiff Metropolitan University, Cardiff, United Kingdom; 2 Department of Electrical Engineering, Faculty of Engineering, German International University, Cairo, Egypt; 3 Department of Electrical and Computer Engineering, Waterloo Engineering School, University of Waterloo, Waterloo, Canada; King Abdulaziz University Faculty of Engineering, SAUDI ARABIA

## Abstract

Developing chaotic systems-on-a-chip is gaining much attention due to its great potential in securing communication, encrypting data, generating random numbers, and more. The digital implementation of chaotic systems strives to achieve high performance in terms of time, speed, complexity, and precision. In this paper, the focus is on developing high-speed Field Programmable Gate Array (FPGA) cores for chaotic systems, exemplified by the Lorenz system. The developed cores correspond to numerical integration techniques that can extend to the equations of the sixth order and at high precision. The investigation comprises a thorough analysis and evaluation of the developed cores according to the algorithm complexity and the achieved precision, hardware area, throughput, power consumption, and maximum operational frequency. Validations are done through simulations and careful comparisons with outstanding closely related work from the recent literature. The results affirm the successful creation of highly efficient sixth-order Lorenz discretizations, achieving a high throughput of 3.39 Gbps with a precision of 16 bits. Additionally, an outstanding throughput of 21.17 Gbps was achieved for the first-order implementation coupled with a high precision of 64 bits. These outcomes set our work as a benchmark for high-performance characteristics, surpassing similar investigations reported in the literature.

## 1 Introduction

**Fig 1:**
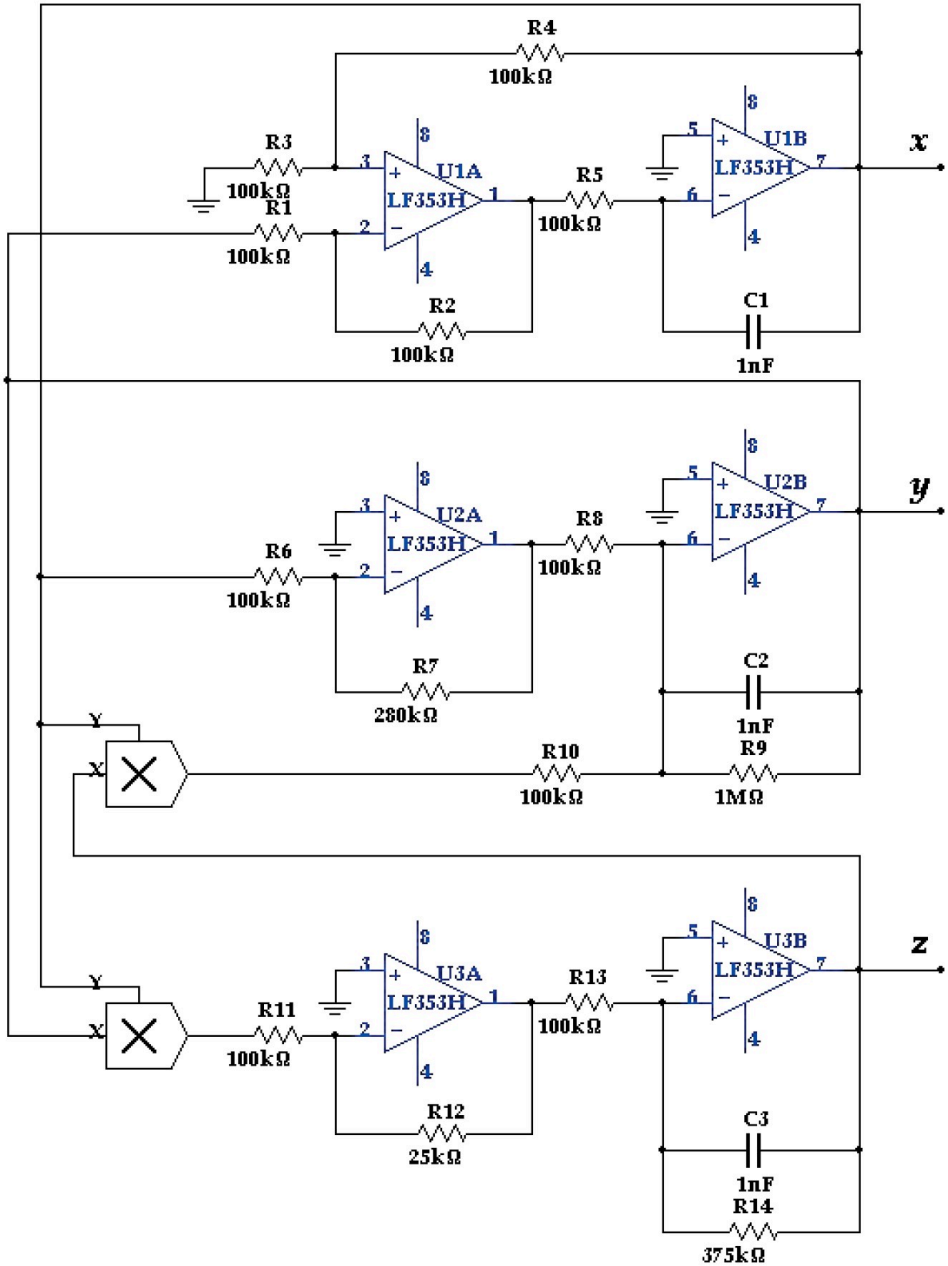
Electronic analogue implementation of the Lorenz system.

**Fig 2:**
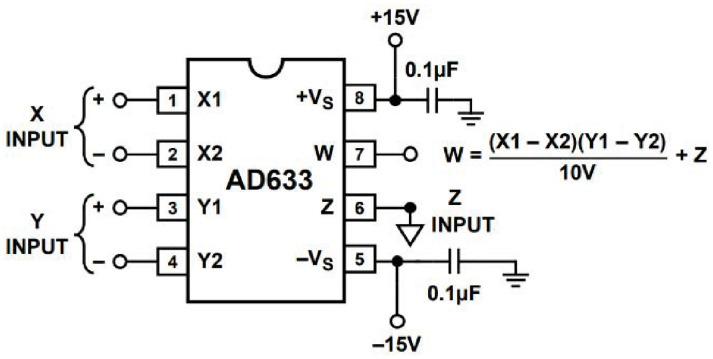
Typical configuration of the AD633 IC, acting as a multiplier.

Many chaotic systems, along with their applications in Chaos-Based Secure Communication (CBSC), data encryption, and True Random Number Generation (TRNG) are implemented using a wide variety of embedded systems, such as Arduino, Application-Specific Integrated Circuits (ASICs), Digital Signal Processors (DSPs), and Field Programmable Gate Arrays (FPGAs) [1–3]. Until recently, and before the rabid advances of digital technology, analogue implementations of continuous-time chaotic, or hyperchaotic, systems were the default. A combination of Op-Amps, resistors, capacitors and analogue multipliers were used to construct such implementations. The Ordinary Differential Equations (ODEs), which are used to describe the dynamics of chaos, for both autonomous and nonautonomous systems, were directly mapped to active RC circuits to generate the states of the system. A typical example, describing the Lorenz system, is illustrated in Fig 1 (see Eq 9) in Section 1). Other examples for analogue implementations could be found in [4–8]. The analogue multiplier AD633 was used to implement the nonlinear part of the Lorenz equation (see Eq (1) in Section 1), along with other chaotic systems of similar structure. As shown in Fig 2, grounding terminals 2, 4, and 6 can effectively produce the product function, with high accuracy.

The Lorenz system was explored in [4, 5], where both the LF353 Op-Amp and the AD633 analogue multiplier were used to perform the required algebraic/calculus-based mathematical operations to implement its dynamics. Adjusting the values of the resistors and the capacitors were used to arrive at the required dominant time constants of the circuit, which could be made as small as a few microseconds, without any noticeable degradation in the performance. Other autonomous chaotic systems, such as the Rössler and Chua circuits, were also considered in [4–6] that covered applications in chaos control, state observers, parameter identification, and synchronization of chaotic systems. Similar analogue implementations to other chaotic systems that include infinitely many equilibria and fractional-order dynamics without equilibrium were also covered in [7, 8].

Challenges to digital implementations of chaotic systems, including Lorenz, include the performance aspects of time, speed, complexity, precision, and dealing with the intrinsic sequential behaviour of the model. As related to chaotic systems, the following research opportunities are highlighted:

- The attraction of reconfigurability of FPGAs in implementing chaotic systems with effective applications in synchronization, control, and communication.
- The development of hardware implementations of chaotic algorithms under FPGAs with appealing performance characteristics that outperform similar implementations reported in the literature.
- The embedding of Lorenz hardware cores to assist or replace traditional computing systems, such as central processing units, in applications.
- The emergence of hybrid analogue and digital chaotic system implementations.
- The exploration of implementations with various accuracy levels, speeds, and complexities.
- The creation of development and analysis patterns that are applicable in the wider area of chaotic systems, such as autonomous and non-autonomous systems to cover both chaotic and hyperchaotic systems.

In this paper, we present high-speed hardware implementations of chaotic systems, namely the Lorenz system. The presented implementations target traditional and high precision including 8, 16, 32, and 64 bits floating point number representations. The proposed hardware cores implement different numerical integration (discretization) techniques that extend to equations of the sixth order. Furthermore, the implementation challenge is extended to include experimenting with different floating-point data types to arrive at the best compromise among complexity, precision, area, and speed.

The rest of this paper is organized so that Section 2 presents related work. Section 3 presents the motivation and research objectives. In Section 4, the adopted hardware development methodology and the created cores are presented. Section 5 presents the achieved results and a thorough evaluation that includes comparisons with closely-related work. In addition, Section 5 presents the design and implementation limitations of the proposed cores and sets the ground for future work. In Section 6, the investigation is concluded by highlighting important achievements and presenting work in progress.

## 2 Related work

### 2.1 Background

When dealing with chaotic systems, several benchmark models exist that can be used for verifying newly proposed techniques, either for control, synchronization, synthesis, or implementation [9]. The Lorenz system is the most famous example that represents the autonomous category of chaotic systems; it has many different forms, including a hyperchaotic model. It was originally discovered when analyzing weather patterns that exhibit very strong dependence on initial conditions [10]; however, other applications in engineering and physics were found to exhibit quite similar behaviour. This includes permanent magnet synchronous machines (PMSMs) [11], single mode optical lasers [12], and thermal convection [13]. The mathematical model of the 3D chaotic Lorenz system is given by Eq 1.

$$\begin{array}{c} \begin{array}{c} \dot{x}=-\sigma (x-y)\\ \dot{y}=\rho x-y-xz\\ \dot{z}=xy-\beta z \end{array} \end{array}$$ (1)

where *x*, *y*, and *z* are the three dynamic states of the system, and *σ*, *ρ*, and *β* are three positive constants. Along with the origin, this system has the two additional equilibrium points of Eq 2.

$$\begin{array}{c} \left[x_{eq}\;\;\;\;y_{eq}\;\;\;\;z_{eq}\right]=\left[\pm \sqrt{\beta (\rho -1)}\;\;\;\;\pm \sqrt{\beta (\rho -1)}\;\;\;\;\left(\rho -1\right)\right] \end{array}$$ (2)

which might be stable or unstable, depending on the values of the parameters, as can be deduced by evaluating the eigenvalues of the Jacobian matrix in Eq 3, at the equilibrium points:

$$\begin{array}{c} J=[\begin{array}{ccc} -\sigma & \sigma & 0\\ \rho -z_{eq} & -1 & -x_{eq}\\ y_{eq} & x_{eq} & -\beta \end{array}] \end{array}$$ (3)

For generating chaos, the parameters might take the values, 10, 28, and 8/3, respectively [9]. The most important characteristics of the Lorenz system are that each dynamic equation contains a single parameter and that chaos is generated by only two quadratic terms; namely, *xy* and *xz*. In addition, it is invariant under the transformation (*x*, *y*) → (−*x*, −*y*). Eq 1 is known to have (0.90563, 0, −14.57219), as Lyapunov exponents, and a DKY of 2.06215, representing the Kaplan-Yorke dimension [14]. Moreover, The Lorenz system is dissipative, as illustrated in Eq 4:

$$\begin{array}{c} \frac{\partial \dot{x}}{\partial x}+\frac{\partial \dot{y}}{\partial y}+\frac{\partial \dot{z}}{\partial z}=tr\left(J\right)=\sigma -1-\beta =-13.667<0 \end{array}$$ (4)

**Fig 3:**
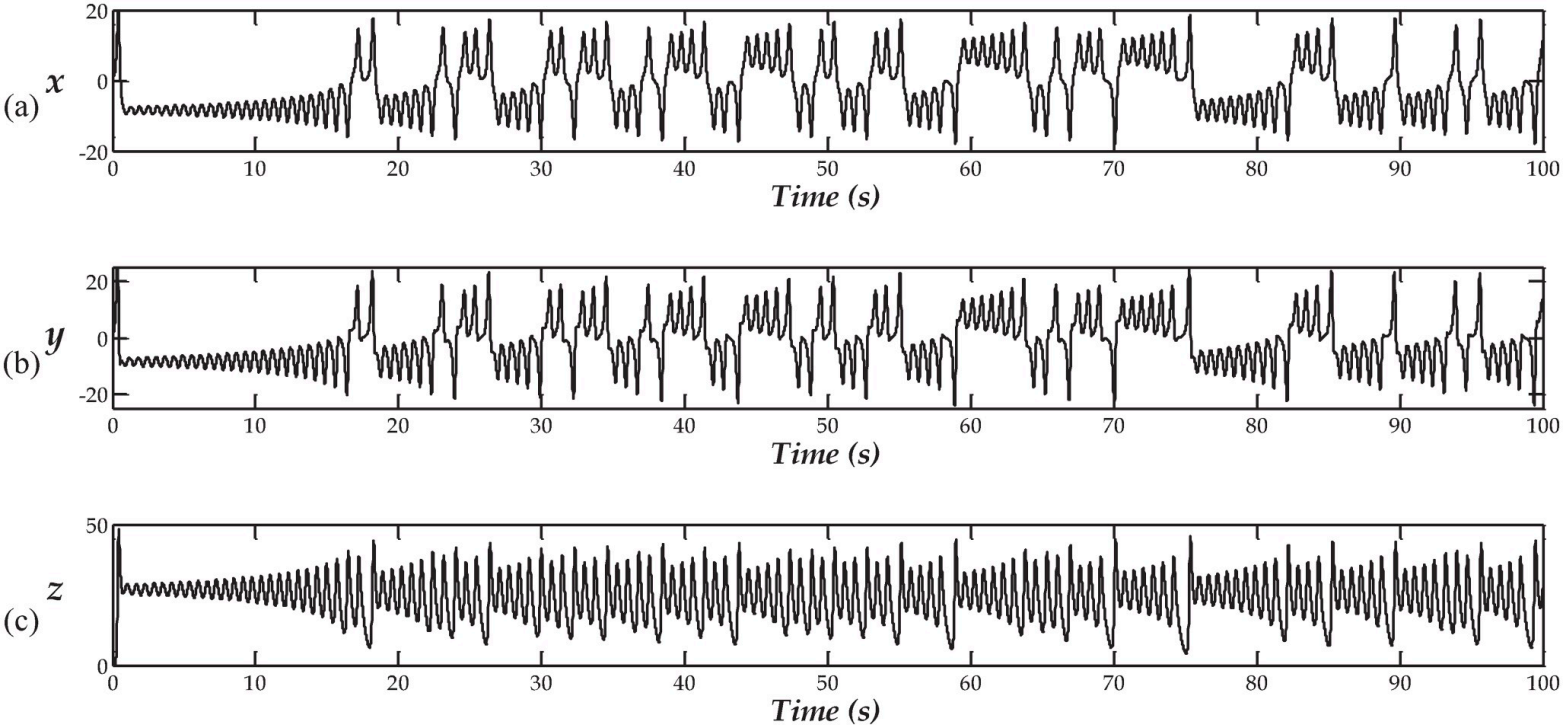
The time series for x(t), y(t), and z(t), in (a), (b), and (c), respectively.

When investigating the time evolution of Eq 1, starting from *x*(0) = 1.0, *y*(0) = *z*(0) = 0, the response, illustrated in Fig 3, is observed.

which is shown to be bounded, for the given values of the parameters. The following ranges for the states were observed, for 50 ≤ *t* ≤ 100:

$$\begin{array}{c} \begin{array}{c} +17.9032\leq x(t)\leq +18.4669\\ -24.0120\leq y(t)\leq +25.0480\\ +04.3772\leq x(t)\leq +45.6160 \end{array} \end{array}$$ (5)

**Fig 4:**
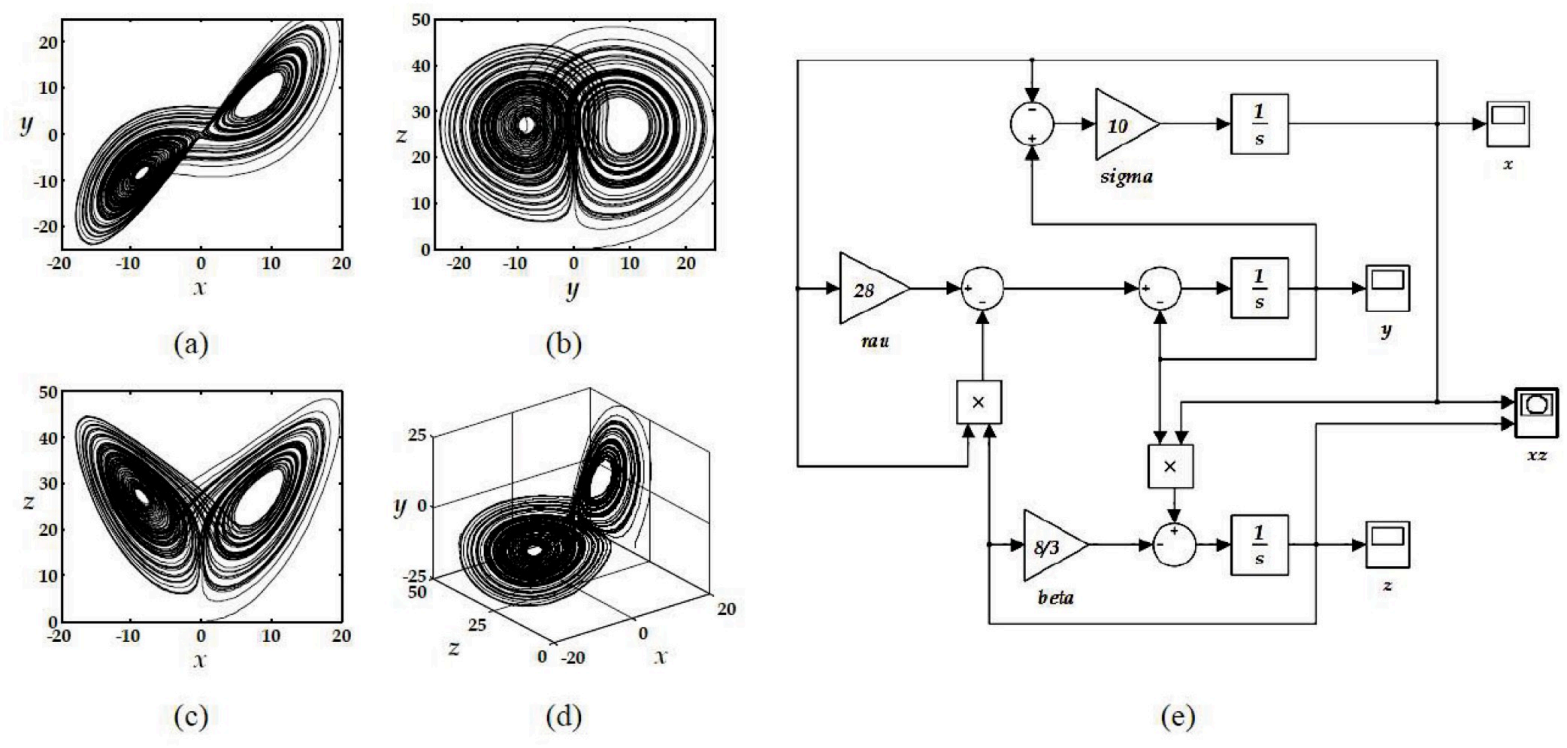
Phase spaces of the Lorenz system in (a)-(d), resulting from simulating the Simulink model.

Examining the phase space of the states, shown in Fig 4, illustrates the chaotic behavior of the system, where the famous butterfly effect is observed. The simulation was conducted employing the Simulink model, as depicted in Fig 4(e). A fixed integration step of 0.01 seconds was maintained throughout the process. Furthermore, the fourth-order Runge-Kutta (RK-4) method was utilized to solve the ODEs presented in Eq 1.

Usually, the choice of the integration step for numerical simulations is based on the actual dominant time constant of the system, in addition to the stiffness ratio of the ODEs [15]. However, for chaotic systems, this is difficult to be extracted from the power spectrum of the states, or the eigenvalues of the Jacobian matrix. Changing the value of *ρ* in Eq 1, while maintaining both *σ* and *β* at their nominal values can lead to different oscillatory non-chaotic patterns that will be stable, provided that the following condition is satisfied [9]:

$$\begin{array}{c} \rho <\sigma (\frac{\sigma +\beta +3}{\sigma -\beta -1})\Rightarrow \rho <\frac{470}{19} \end{array}$$ (6)

which is directly driven from the eigenvalues (λ_*i*_, *i* ∈ 1,2,3) of the characteristics equation that corresponds to Eq 3. In addition, the eigenvalues of Eq 3, at the nominal values of *σ*, *ρ*, *β* and the equilibrium points of Eq 2 can be used to calculate the stiffness ratio (SR), as depicted in Eq 7:

$$\begin{array}{c} \lambda =[\begin{array}{c} -13.8546\\ 0.0940+j10.1945\\ 0.0940-j10.1945 \end{array}]\Rightarrow SR=\frac{13.8546}{0.0940}=147.39 \end{array}$$ (7)

The SR, calculated in Eq 7, which is the ratio of the largest to the smallest eigenvalue of the Jacobian matrix of the ODE system, depicted in Eq 3, has a large value reflecting more restrictive stability conditions for the Lorenz system. This signifies that the solution, despite varying slowly, is affected by other nearby solutions that vary rapidly, so the chosen numerical method must take small integration steps to obtain satisfactory results. This should be taken into consideration, when designing the FPGA-based numerical algorithm, in terms of the maximum operating frequency, and the solver structure, which is thoroughly analyzed in the coming sections.

**Fig 5:**
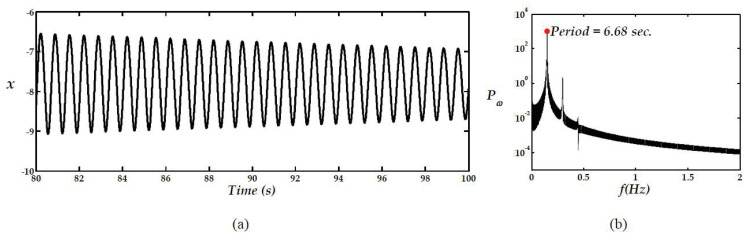
The power spectrum, in (b), for *x*(*t*), in (a), showing a dominant period of approximately 6.68 s.

Fig 5 shows the signal *x*(*t*), for *ρ* = 24, while the remaining parameters are kept the same, along with its power spectrum in (a) and (b), respectively. The periodic time of the dominant cycle can be used as a guide for the best choice of the integration step of the numerical solver.

**Fig 6:**
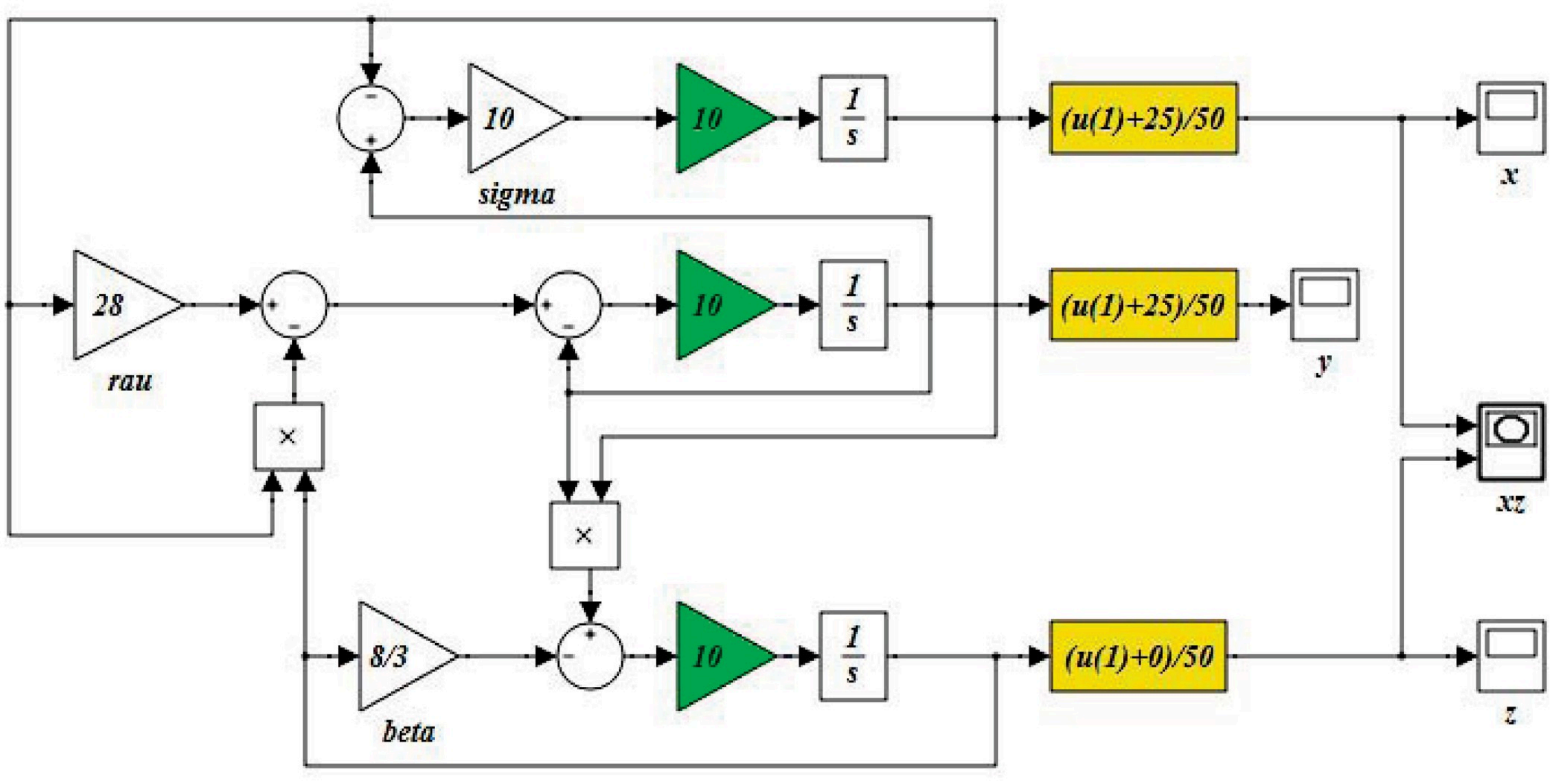
New layout of the Lorenz system with both time and magnitude scaling.

When using the Lorenz system for practical implementations, e.g. CBSC [4], it might be required to scale the generated signals to meet the constraints imposed by the actual hardware. For example, when using standard TTL hardware, signals are required to be within 5 Volts limits. In addition, many ADDA cards require the analogue signals to be within ±10 Volts. Nowadays, many low-power hardware, e.g. modern FPGAs, require dealing with signals that are limited to 3.3 Volts. More restrictions could be imposed on the level of the signals, generated from the Lorenz system, for specific applications that require handling binary-based multimedia signals, corresponding to text, audio, images, and video streams [16]. Consequently, scaling the values of *x*(*t*), *y*(*t*), and *z*(*t*), to meet the required range, should be provided in a systematic way that will not distort the chaotic behaviour of the Lorenz system. Along with magnitude scaling, adjusting the time scale of the Lorenz system might be required to meet the requirements on the bandwidth of the application. This is crucial, especially for real-time applications that require synchronizing the speed of the Lorenz system with some clock. A simple way to achieve scaling, in both magnitude and time, is to modify the Simulink block diagram, as illustrated in Fig 6. The system is made 10 times faster while forcing all states to fall between 0 and 1. This was easily adjusted by adding the gain blocks, just before the integrators (shown in green), while using soft functions to scale all the variables (shown in yellow).

The numerical technique, illustrated in Fig 6 can also be augmented to Eq 1 to yield the following:

$$\begin{array}{c} {}[\begin{array}{c} \dot{x}\\ \dot{y}\\ \dot{z} \end{array}]=T_{SF}\;\text{x}\;[\begin{array}{c} -\sigma (x-y)\\ \rho x-y-xz\\ xy-\beta z \end{array}]\;\&\;\frac{S^{new}-S_{min}^{new}}{S^{old}-S_{min}^{old}}=\frac{S_{max}^{new}-S_{min}^{new}}{S_{max}^{old}-S_{min}^{old}} \end{array}$$ (8)

where *T*_*SF*_ is the time scale factor that is used to shrink or stretch the time if set to more than or less than one, respectively. In addition, the old signal, *S*^*old*^, corresponding to *x*, *y*, or *z* could be easily scaled to *S*^*new*^, for any given range, according to the mathematical expression in Eq 8.

When depending on numerical simulations to generate the chaotic signals, the choice of the integration algorithm and its corresponding time-step is crucial. Numerical solvers convert the analogue model, implicitly, into an equivalent discrete model for which the accuracy is dependent on its order. Stability, convergence, and tolerance are three important factors that must be taken into consideration when choosing the numerical solver and adjusting its settings. Many software packages exist that can do this automatically, e.g. MATLAB. The accuracy of the simulation is directly proportional to the order of the integration algorithm. First-order Euler, second-order Heun, and RK-4 methods are the most famous numerical solvers to choose from. Low-order numerical solvers are simpler, faster and require less mathematical effort, when implemented in real-time embedded hardware. On the other hand, higher-order numerical solvers are more complicated, require access to many intermediate variables, and can be dramatically slow, which makes them less appealing for real-time applications. Thus, an optimal compromise should be obtained between the required details for the abstract level of the discrete-equivalent model and its operating speed. Usually, there is a conflict between accuracy and speed, and satisfying both of them requires very sophisticated hardware with high-performance computational power. As a rule of thumb, the approximation error between the numerical solution and the exact solution is a function of *h*^*n*^, where *h* is the integration step and *n* is the order of the numerical solver. This implies that for better accuracy smaller integration steps and higher-order solvers should be used.

**Fig 7:**
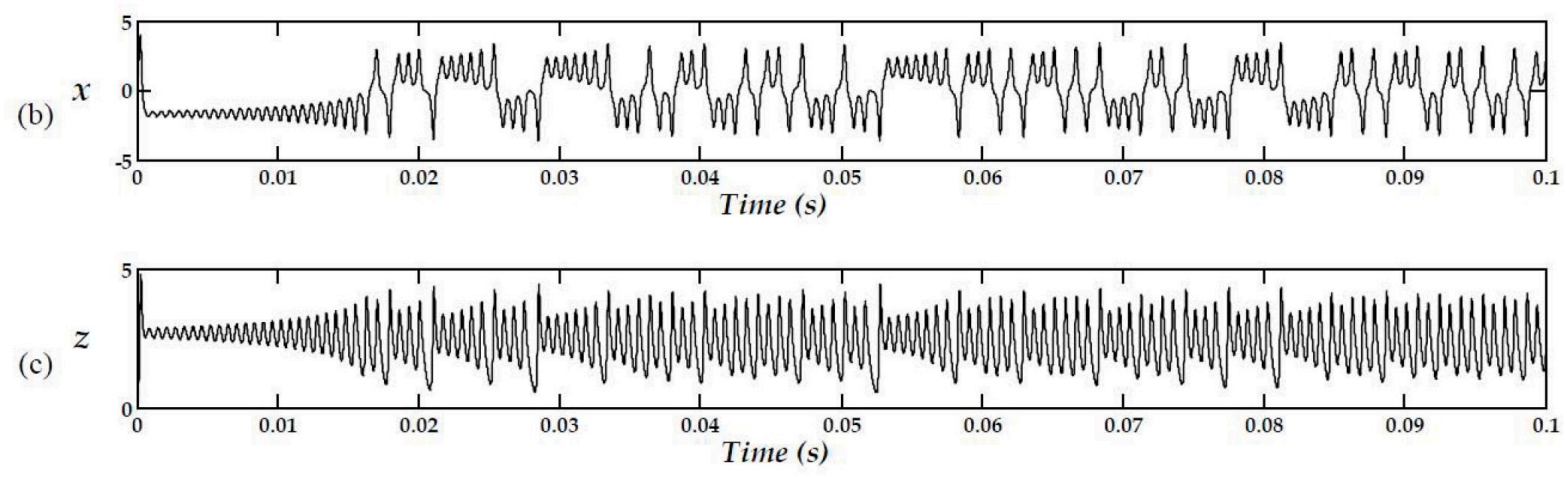
The response of the modified Lorenz system, corresponding to Eq 9.

For many applications, the Lorenz system needs to be implemented in analogue forms, especially in both electronic and optical hardware. In such cases, proper connections should be set up in the laboratories, with a controlled environment to minimize the effects of noise and external disturbances. Analog components are inherently susceptible to degradation over time, influenced by factors such as aging, temperature variations, and additional anomalies that may arise during the circuit assembly process. Therefore, their accuracy might be questioned, and they will need continuous calibration and conditioning. Fig 1 illustrates a typical electronic layout for an analogue implementation of the Lorenz system that has a scaling factor of 1000, and all the signals are scaled to fit the standard TTL level of ±5 Volts [17]. Analog Op-Amps and a collection of resistors and capacitors are used to represent the three first-order nonlinear dynamics of *x*(*t*), *y*(*t*), and *z*(*t*). Two analog multipliers, AD633AN, were used to generate the quadratic terms *xy* and *xz*, while using LF353 Op-Amps, with ±15 Volts power supplies. The values and types of the analog components are shown in Fig 1. The modified ODEs, representing the electronic circuit of Fig 6, are given by:

$$\begin{array}{c} \left[\begin{array}{c} \dot{x}\\ \dot{y}\\ \dot{z} \end{array}]=[\begin{array}{c} \frac{1}{R_{5}C_{1}}(\frac{-R_{3}}{R_{3}+R_{4}}[1+\frac{R_{2}}{R_{1}}]x+\frac{R_{2}}{R_{1}}y)\\ \frac{1}{C_{2}}(\frac{R_{7}}{R_{6}R_{8}}x-\frac{1}{R_{9}}y-\frac{1}{R_{10}}xz)\\ \frac{1}{C_{3}}(\frac{R_{12}}{R_{11}R_{13}}xy-\frac{1}{R_{14}}z) \end{array}]=1000[\begin{array}{c} -10(x-y)\\ 28x-y-10xz\\ 2.5xy-2.667z \end{array}\right] \end{array}$$ (9)

which have a scaling time factor of 1000, a 20% scaling factor for both *x* and *y*, and a 10% scaling factor for *z*. Fig 7 illustrates the response of such system.

The Lorenz system was first observed in an application in fluid convection, where *x*(*t*) represents the rate of the fluid convection, while both *y*(*t*) and *z*(*t*) represent the temperature variation in both the horizontal and the vertical directions. The parameters *σ*, *ρ*, *β* represent Prandtl number, Rayleigh number, and horizontal wave number of the fluid convection, respectively [10]. However, many optical systems have similar dynamics; this suggests the possibility of implementing the Lorenz system using optical devices, in contrast to the previous electronic analogue implementation. Eq 10 exemplifies the dynamics of semiconductor lasers:

$$\begin{array}{c} \begin{array}{c} \dot{x}=-\sigma (x-y)\\ \dot{y}=\rho x-(1-j\delta )y-xz\\ \dot{z}=Re[x^{*}y]-\beta z \end{array} \end{array}$$ (10)

where *σ* represents the decay rate of the electric field, *δ* is the atomic detuning, *ρ* is the pump parameter, and *β* is the decay rate of the population inversion. Now, *x*(*t*), *y*(*t*) and *z*(*t*) are normalized variables that represent the electric field, the polarization, and the population inversion, respectively. With optical implementations, higher-speed applications could be easily addressed. However, laser-based analogue implementations are much more expensive than electronic ones and require special labs to be set up.

With the rapid advancement of digital technology and the current availability of high-performance computing powers, digital implementations of chaotic systems are becoming more feasible and are replacing their analogue counterparts in many applications, especially in CBSC systems that rely on cryptography. This paper addresses the optimization of FPGA-based implementations of chaotic systems. Without loss of generality, only the Lorenz system will be discussed; however, it is argued that extending the suggested techniques is straightforward and very systematic when applied to other chaotic systems with different structures.

### 2.2 Literature survey

Due to the inherent problems in analogue circuits, specifically tolerance of the components, ageing, noise sensitivity, and limited operating bandwidths, digital implementations using discrete-equivalent models are much preferred, especially after the incredibly fast drop in the cost of digital circuitry. Many numerical methods were used to convert the differential equations, corresponding to the continuous-time chaotic systems, into closely equivalent difference equations [18]. This has the effect of converting the complex calculus-based calculations that don’t have closed-form analytical solutions into much easier algebraic-based recursive calculations that are much suited to numerical techniques, using different programming languages and different digital platforms. The one-step numerical algorithms such as Euler, Heun, and Runge-Kutta (RK) methods, in addition to the multi-step algorithms, such as Adams-Bashforth and Adams-Moulton methods, are among the most famous choices, depending on the nature of the system, its stiffness, and whether it is integer or fractional order [19]. Microcontrollers, as a low-cost choice for implementing the discretized chaotic Lorenz system were explored in [20], where the Euler algorithm, with an integration step of 4.0 ms was used. An 8-bit PIC18F452 microcontroller was used, with a clock frequency of 10 MHz, while coding the algorithm using a CCS-C compiler. It was argued that the adopted implementation is much cheaper than using an FPGA approach; each run needed 350 *μ*s, while 6% and 9% of the allocated RAM and ROM were used, respectively. Another choice for digital implementation of chaotic systems was adopted in [21], using a 32-bit TMS320F28335 DSP board running at 150 MHz, with floating point arithmetic operations, along with the 16-bit DAC8552, connected through a serial peripheral interface. This DSP-based system used the RK-4 numerical solver, with an integration step of 1.0 ms, to analyze the behavior of Chua system, with a hidden attractor. It was found that the experimental results are in good agreement with the MATLAB-based simulation results. Other approaches to digitally design and implement discretized chaotic systems were explored in [22–24] to address software techniques that work with and without MATLAB/Simulink engine, the use of ASICs versus FPGAs, and LabVIEW-based FPGAs, respectively.

In addition to the analogue implementations of different chaotic systems that were explored in [4–8, 19, 24], more examples were presented in [25–28] to compare their performance to that of an equivalent FPGA-based implementation. In [25], a comparison was made between an analogue simulation-based and FPGA-based implementations, for a new chaotic system with a single equilibrium point. The analogue circuit was constructed using Pspice, while a Xilinx Virtex-6 family xc6vlx75t-3ff784 FPGA was used for the digital implementation. Adopting both the Heun and the RK-4 algorithm resulted in a maximum frequency (Fmax) of 390.067 MHz, using a 32-byte IEEE 754-1985 floating point numerical format for the VHDL code. Based on the reported results, the generated data were consistent with a convergence of 34.456E-5 precision, using absolute error analysis. In [26], a similar study was conducted, but for a chaotic TRNG that is based on the Sundarapandian–Pehlivan system. Signals were generated from an actual analogue circuit implementation that was initially modelled and tested using Pspice, and then compared to a digital implementation using a Xilinx Virtex-6 XC6VLX240T-1-FF1156 chip that adopted RK-4, as the discretization method. The digital implementation used the high precision 32-bit IEEE 754-1985 standard and managed to achieve a Fmax of 293.815 MHz. Moreover, the superiority of the FPGA-based implementation was verified by passing the two popular statistical-based standards, FIPS-140-1 and NIST-800-22, which proves their suitability for cryptographic applications.

Another study for digitally implementing a TRNG that depends on the generalized Sprott C chaotic system was developed in [27]. Although the system under study could exhibit multi-butterfly chaotic attractors, a comparison was made for the case of generating a two-butterfly chaotic attractor only. A discretized Euler-based method was used, with an integration step of 1 ms, and the FPGA-based hardware was a Xilinx DSP System Generator. The throughput of the digital implementation was analyzed, and power consumption was reported. Again, both the analogue and the digital results were consistent, and the designed system was able to pass 16 runs in the NIST-800-22 standard test. Another comparison between a Multisim-based simulation model and an FPGA-based model was conducted in [28], for a 3-D multi-stable system with a peanut-shaped equilibrium curve that was used for an image encryption application. The used FPGA was a Cyclone IV, with a 50 MHz clock and Quartus II synthesizer. Three different discretization methods were used, Euler, Trapezoidal, and RK-4, with an integration step of 0.1 ms. All of them were found in perfect agreement with the results obtained from the Multisim model. These different FPGA-based examples that were applied to many different chaotic systems and span many applications were found very effective. The choice of the discretization algorithm, deciding on the integration step, and achieving the highest frequency for real-time operation, along with other important factors related to the throughput of the FPGA-based digital implementation need to be carefully analyzed in order to ensure the integrity of the obtained results and their consistency with their analogue counterparts.

## 3 Research objectives

The proposed investigation aims at achieving several research objectives. The investigation focuses on developing high-speed FPGA cores for chaotic systems as exemplified by the famous Lorenz system. The proposed developments are set to challenge state-of-the-art FPGAs by targeting numerical integration techniques that can extend to equations of the sixth order. Furthermore, the implementation challenge is extended to include experimenting with different floating-point data types to arrive at the best compromise among complexity, precision, area and speed. The proposed implementations include high-order equations and high-precision floating point representations that are limitedly addressed in the literature. Indeed, the proposed investigation presents a development pattern that can be adopted in the wider area of chaotic systems. As the developments comprise challenging implementations, the investigation presents an analysis pattern that can be adopted for other chaotic systems. The investigation presents a thorough discussion and comparison among analogue, software, and hardware implementations under FPGAs. The proposed developments enable discussing the extendibility of the investigation to applications, such as CBSC. The research objectives of this paper are summarized as follows:

1. Develop high-speed FPGA cores for Lorenz chaotic systems with discretizations of the first, fourth, and the sixth order.
2. Target implementations with different precision, namely, 8-bit, 16-bit, 32-bit, and 64-bit floating-point numbers.
3. Perform a thorough analysis of the developed cores per complexity, power consumption, precision, area, throughput, and maximum operational frequency.
4. Validate the findings through careful comparisons with outstanding closely-related work from the recent literature.
5. Discuss the limitations of the proposed work and set the ground for future work.

In relation to the similar work presented in Section 2, the proposed development enables the following comparisons for all the implemented cores. The pattern of comparisons includes reasoning about the development methodology and the target performance goals. In addition, the comparison presents a focus on the achieved precision per algorithm with and without scaling:

- Evaluation of the attained maximum frequency.
- Evaluation of the attained throughput.
- Evaluation of the attained hardware area in terms of logic elements and registers.
- Evaluation of the attained power consumption.

The investigation confirmed the successful achievement of high-speed and accurate FPGA cores that outperform similar work reported in the literature in several aspects.

## 4 Hardware design

An informal and systematic approach is adopted to develop hardware cores for the targeted Lorenz system [29, 30]. The methodology is unified in the sense that it uses common software engineering techniques to model the algorithm; accordingly, HW and *SW* designs are derived and implemented. The steps of the HW and SW developments are as follows:

1. Depict the algorithm using flowcharts.
2. Develop the software version.
3. Capture the parallelism in the algorithm using concurrent process models.
4. Design the processor Datapath by identifying, allocating, and binding resources.
5. Develop the Finite State Machine (FSM) of the control unit based on the flowchart.
6. Describe the developed hardware using a description language and synthesize the implementation for FPGAs.

**Fig 8:**
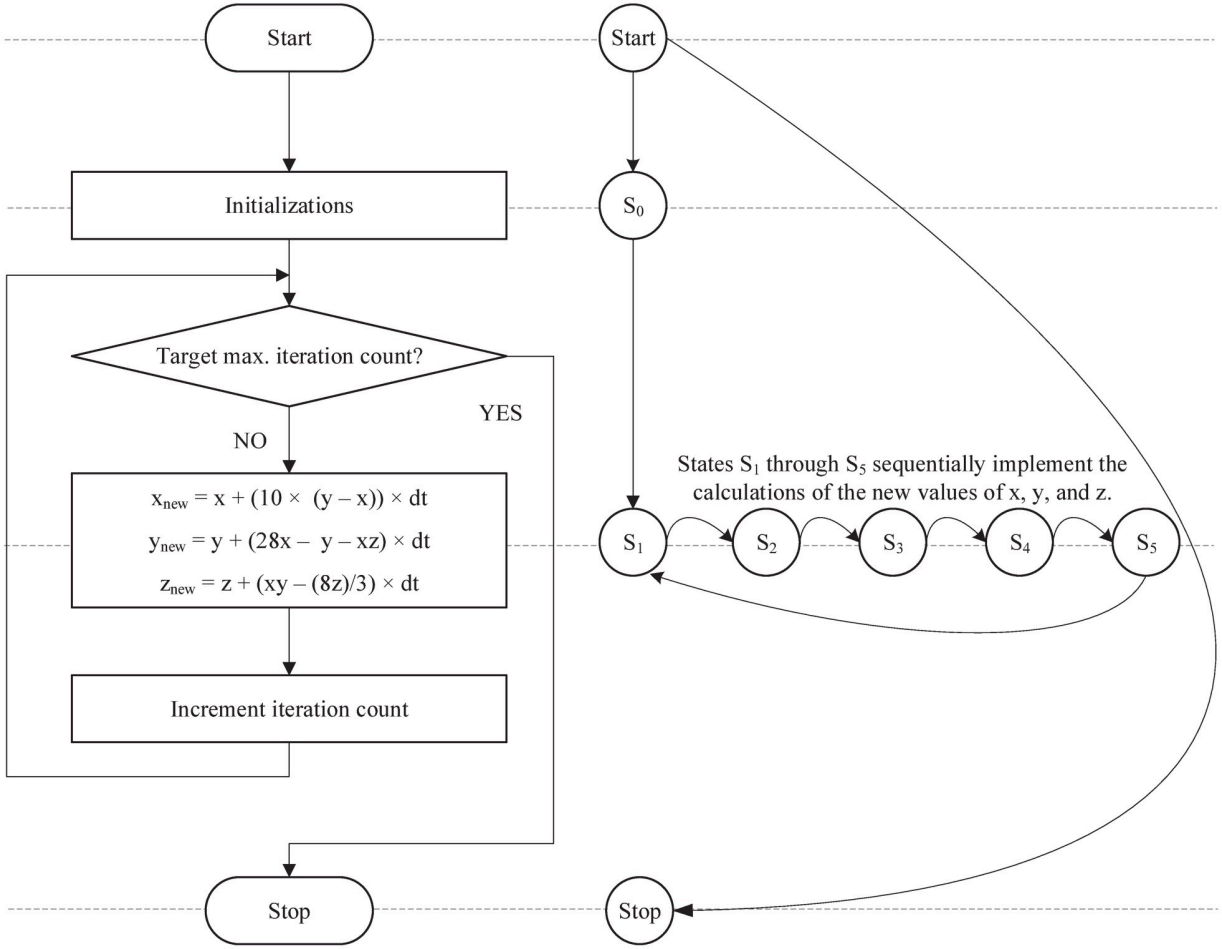
The flowchart and FSM of Euler discretization algorithm.

Fig 8 lays out the conceptual behaviour of the first-order Lorenz system, capturing the flow of the algorithm along with the states that the system evolves through to attain the desired output. The aim behind our proposed hardware core is to compute the different values of $\dot{x}$, $\dot{y}$, and $\dot{z}$ that vary over time by solving a set of differential equations expressed in Eq 1. Those computations are carried out repeatedly until a target number of iterations is reached.

**Fig 9:**
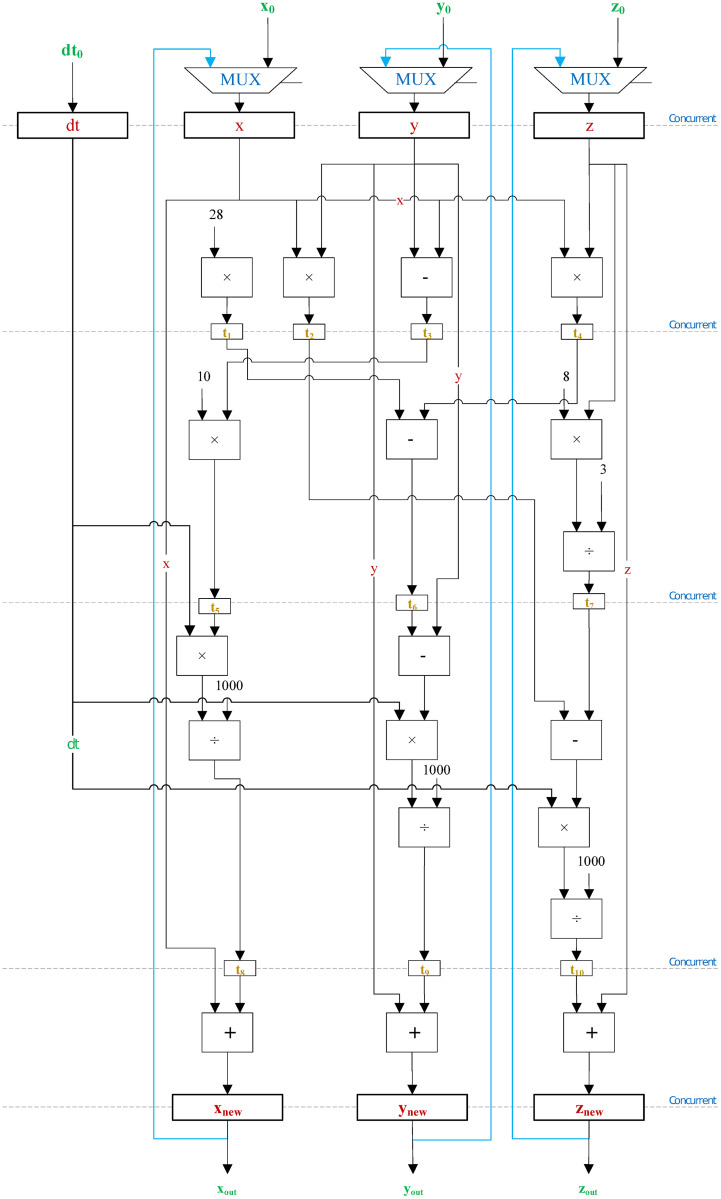
The datapath of Euler discretization algorithm.

Inspired by the electronic analogue implementation of the Lorenz system presented in Fig 1, several computational hardware resources are allocated to develop the datapath of Lorenz’s digital model as shown in Fig 9. Our main focus in the proposed algorithm is to utilize floating-point (FP) functional units to execute the required arithmetic operations for solving the aforementioned set of differential equations. To facilitate the process of hardware development in VHDL, FP computational units are imported from off-the-shelf IEEE libraries. Those units include adders, subtractors, multipliers, and dividers. In addition, multiplexers and registers are employed to load and store various sets of data at different intervals of time. It is important to note that the datapath presented in Fig 9 is for the Euler discretization algorithm. However, by following the same design methodology, and by utilizing additional hardware resources, this model can easily be upgraded to solve higher-order differential equations including RK-4 and RK-6 algorithms.

Hardware designers are usually confronted with a multitude of challenges when it comes to designing effective hardware cores that comply with the requirements of a real-time system. Among these challenges are, maximizing the processor’s frequency, diminishing the period of each cycle, activating concurrent utilization of different hardware resources, and many more. To this end, our proposed algorithm described in Listing 1 is partitioned into 6 states from *S*_0_ to *S*_5_ as presented in Fig 8. Those states depict the behaviour of the control unit at different intervals of time when certain conditions are met. State *S*_0_ is responsible for initializing the values of all the registers simultaneously. States *S*_1_ through *S*_5_ each are responsible for the parallel execution of independent arithmetic computations by the concurrent utilization of FP functional units; after which the resulting values are stored in temporary registers to be used in the coming states. This design approach provides an efficient hardware utilization scheme and attains phenomenal results when operating in real-time.

**Listing 1**. Sample VHLD code segment for the Euler discretization algorithm entity showing the main computational resources

…

**architecture** behavioral **of** EulerAlgorithm **is**

…

**begin**

…

**process**(current_state)

**begin**

 **case** current_state **is**

  **when** Sreset=>

   update <= ‘0’ ;

   next_state <= S0 ;

    **when** S0 =>

        x <= xi ;

        y <= yi ;

        z <= zi ;

        dt <= dti ;

        update <= ‘0’ ;

        next_state <= S1 ;

    **when** S1 =>

        t1 <= 28*x ;

        t2 <= x*y ;

        t3 <= y-x ;

        t4 <= x*z ;

        update <= ‘0’ ;

        next_state <= S2 ;

    **when** S2 =>

        t5 <= (10 * t3) ;

        t6 <= (-t4 + t1) ;

        t7 <=((8 * z) / 3) ;

        next_state <= S3 ;

    **when** S3 =>

        t8 <= ((t5 * dt)/1000) ;

        t9 <= (((t6 − y) * dt)/1000) ;

        t10 <= (((t2 − t7)*dt)/1000) ;

        next_state <= S4 ;

    **when** S4 =>

        xnew <= x + t8 ;

        ynew <= y + t9 ;

        znew <= z + t10 ;

        next_state <= S5 ;

    **when** S5 =>

        x <= xnew ;

        y <= ynew ;

        z <= znew ;

        xout <= xnew ;

        yout <= ynew ;

        zout <= znew ;

        update <= ‘1’ ;

        next_state <= S1 ;

    …

**end case** ;

**end process** ;

**end architecture** ;

## 5 Analysis and evaluation

In this section, a thorough analysis and evaluation are presented of the variety of developed cores. Firstly, the results are presented highlighting some achieved appealing performance characteristics. Secondly, the results are evaluated with a focus on the practical implications and achievements in both the general application and specific technical aspects. At that point, comparisons with multiple closely-related investigations are presented. The section ends by identifying limitations and proposing future research directions.

### 5.1 Results

**Fig 10:**
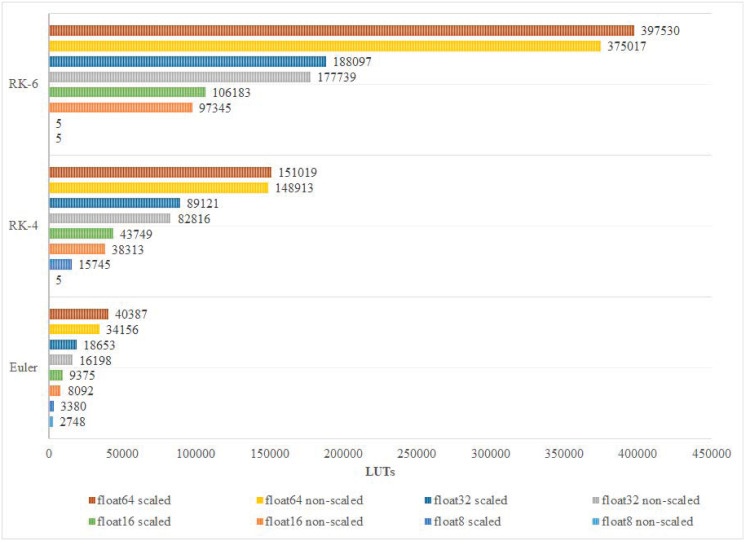
Number of utilized LUTs classification.

**Fig 11:**
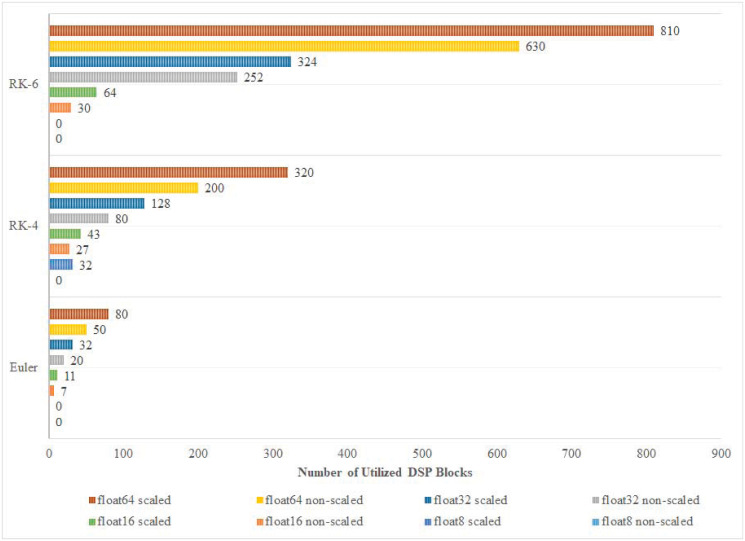
Number of utilized DSP blocks classification.

**Fig 12:**
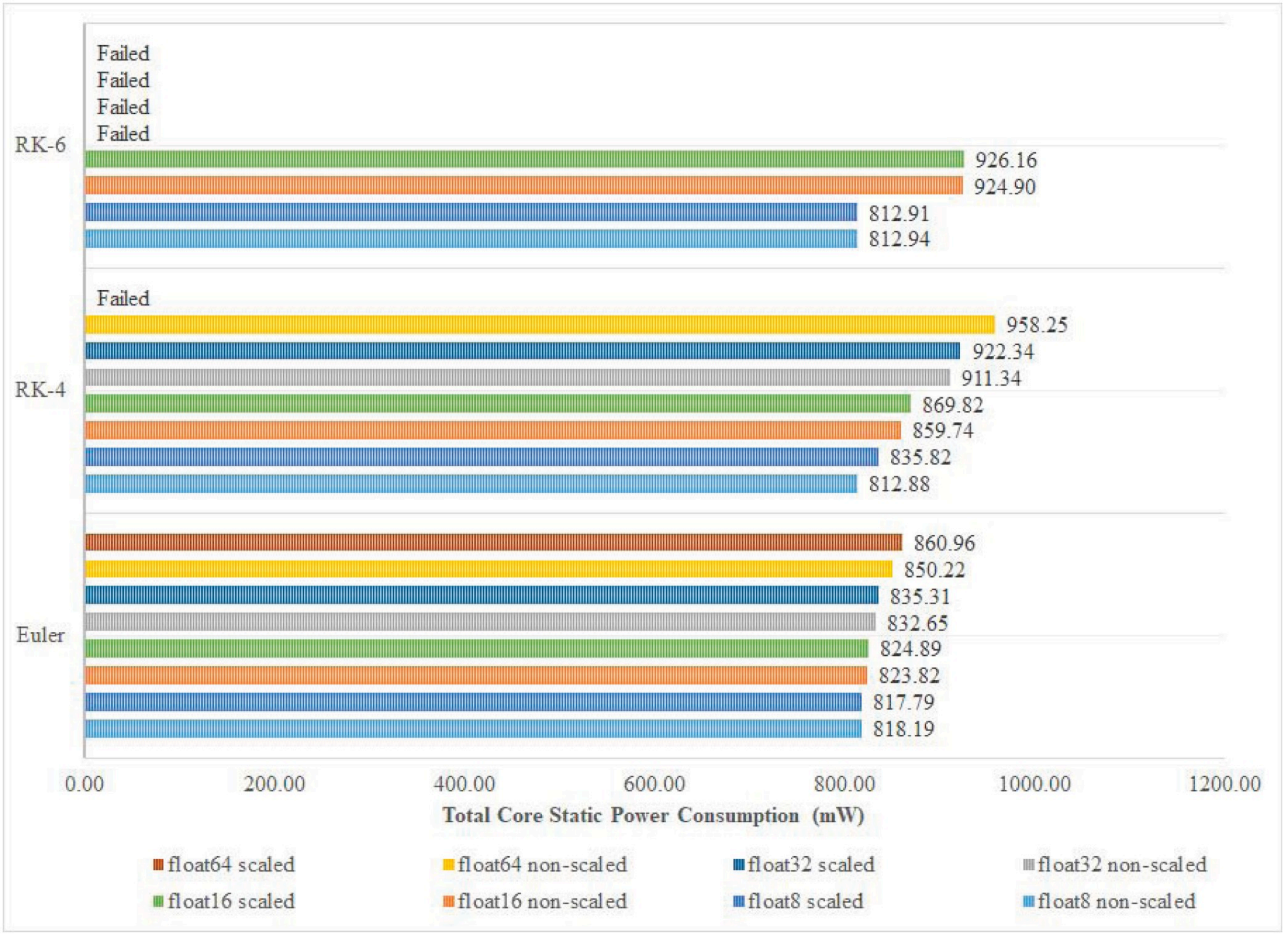
Total power consumption classification.

In this paper, scaled and non-scaled implementations of Euler, RK-4, and RK-6 discretization algorithms are presented. The results confirm that, in most cases, complex implementations that are scaled and have higher precision, and discretization algorithm order, utilize more DSPs and LUTs than simpler non-scaled implementations as shown in Figs 10 and 11. However, this does not hold true in some special cases. For instance, the number of utilized LUTs in the 8-bit Euler algorithm is 2,748 which is more than that of the 8-bit RK-4 system which is 5 LUTs. The reason behind this variation is that, at compilation, the synthesizer may detect an optimization opportunity that only can be carried out on the higher-order system that bears more hardware units than the lower-order system. As for Logic Registers (LRs), Euler, RK-4, and RK-6 algorithms utilize 7, 12, and 16 LRs respectively, regardless of the adopted floating point precision in each implementation. Fig 12 shows how power consumption follows the trend of hardware utilization, expressing how different designs consume more power upon utilizing additional hardware resources. It is important to note that while experimenting with implementations of different configurations, some of them failed to compile. Such failures occur when the device under testing do not possess the minimum number of hardware resources that a certain hardware design demands.

**Fig 13:**
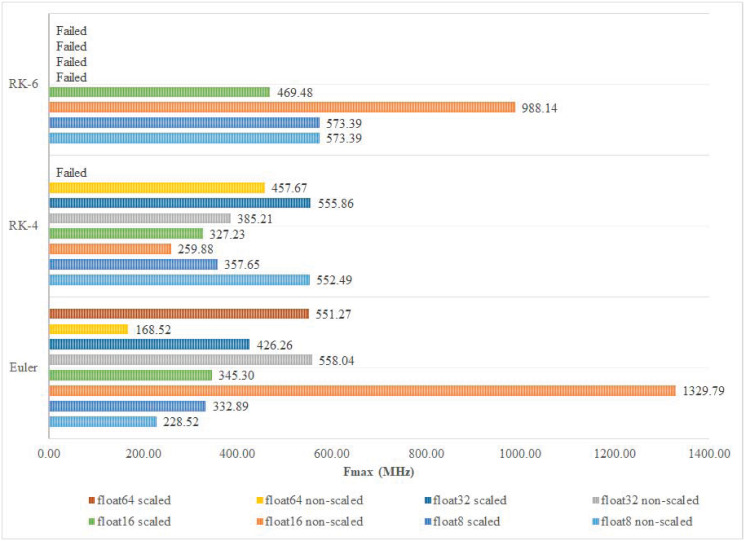
Fmax classification.

**Fig 14:**
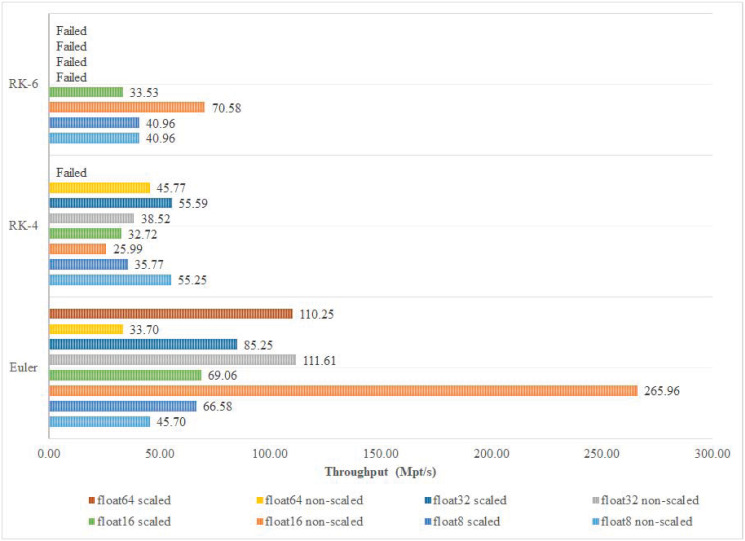
Throughput (Mpt/s) classification.

**Fig 15:**
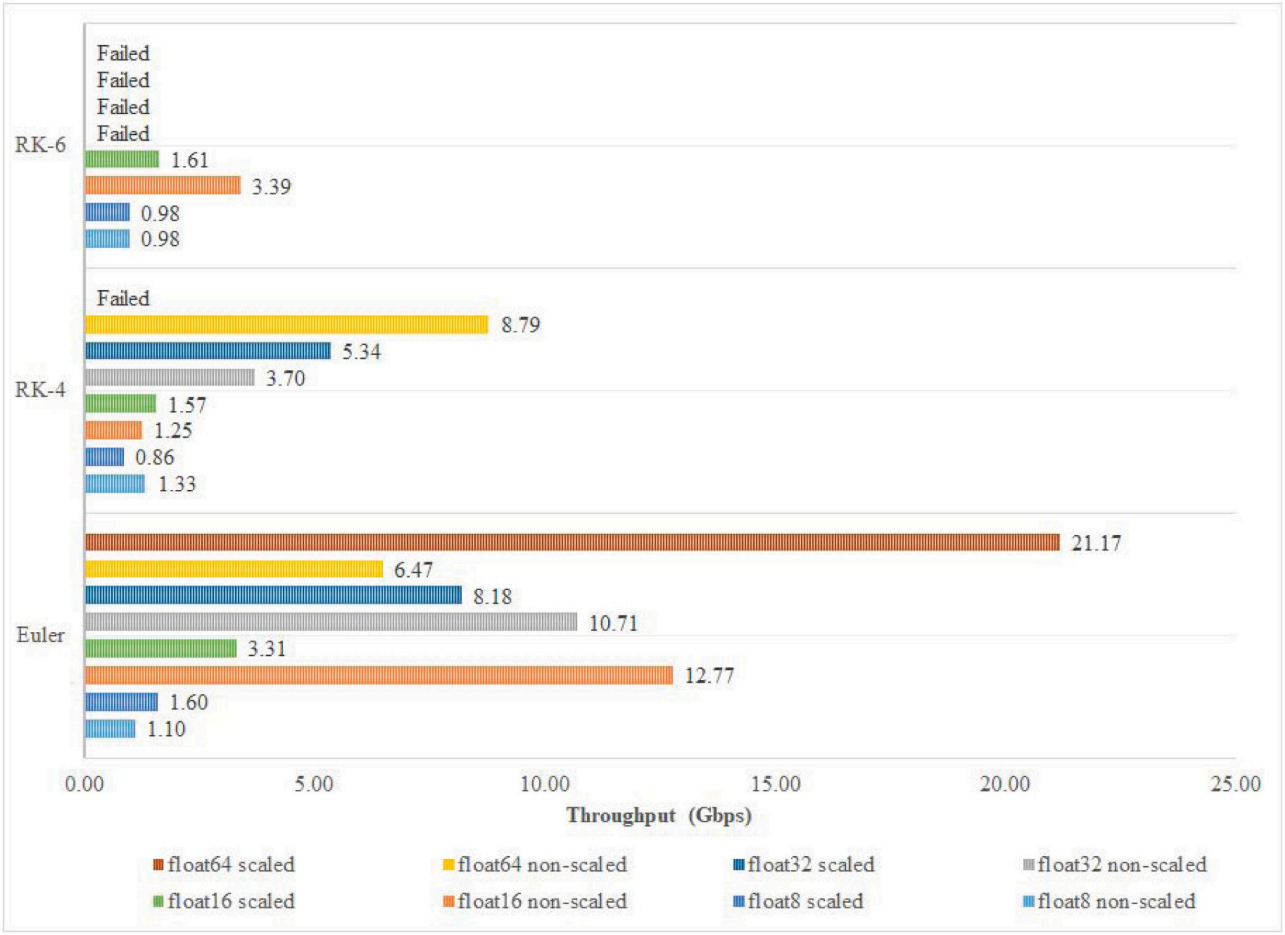
Throughput (Gbps) classification.

To better understand the effect of FP precision and discretization algorithm on the system’s performance, Fmax, throughput in Gbps, and throughput in Mpt/s are recorded for each implementation. Among the different implementations of Euler and RK-6 algorithms, the 16-bit non-scaled version achieves the highest operating frequency and highest throughput in Mpt/s as shown in Figs 13 and 14 respectively. Those results are not fully maintained by the Euler algorithm when it comes to throughput in Gbps, where the 16-bit non-scaled version attains a throughput of 12.77 Gbps which is topped by the 64-bit scaled version that attains a throughput of 21.17 Gbps. However, in the RK-4 algorithm, the 32-bit scaled version achieves the highest operating frequency of 555.86 MHz and throughput of 55.59 Mpt/s, while the 64-bit non-scaled version achieves the best throughput in Gbps, operating at a rate of 8.79 Gbps as shown in Fig 15.

**Fig 16:**
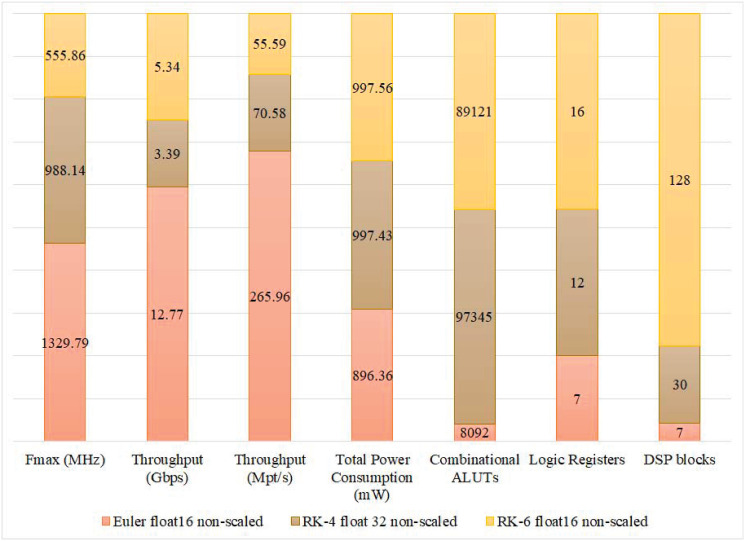
The performance indicators vector of the different discretization algorithms that attained the highest throughput.

Fig 16 presents the best-achieved results per discretization algorithm order. The 16-bit non-scaled version proved to be superior to other implementations in Euler and RK-6 algorithms. However, in the RK-4 algorithm, the aforementioned implementation had the worst performance as opposed to the 32-bit scaled version that stood out among other implementations, achieving significant performance results as shown in Fig 16.

### 5.2 Evaluation

Discretization of continuous-time systems is a numerical approximation that needs to faithfully replicate the original behaviour of the system. The discretization algorithm and the used integration step are the most important factors in arriving at the required accuracy. For simple one-step discretization, using the Euler method, there is a strong need to use a very small integration step to avoid the accumulation of residual errors. When adopting a higher number of the intermediate steps in the discretization method (e.g. RK-4 and RK-6), a relaxation could be made to the choice of the minimum integration step. Indeed, this comes at the expense of the mathematical complexity and the required computing power. To avoid the accumulation of roundoff errors, especially for real-time applications that require continuous operation, a higher precision for data representation should be used. With the rapid advances in digital technology and the current availability of configurable hardware, this became readily available. In this paper, we addressed the RK-6 algorithm, with an outstanding increased accuracy, which is indeed a major contribution, as all the work reported in the literature relies mainly on RK-4. The RK-6 algorithm will prove more stable, robust, and rigorous for real-time applications, especially those that require hyperchaotic systems. Moreover, the designed discretization algorithms in this paper were able to accommodate different operating conditions, via providing easy scaling of operating frequency and range of the output values to suit different digital hardware requirements, e.g. the new 3.3 V FPGAs. This added flexibility required little overhead in the implementation, which makes them suitable candidates for different applications in the field of CBSC and TRNG.

Developing digital hardware implementations of chaotic systems is driven by different solid motivations. The widespread analogue implementations, the nature of the utilized computations, and the appealing pipeline-like structure are among the important attractions for hardware developments. Chaotic systems discretization methods are constructed using fine-grained computational building blocks that with no doubt can promise high performance if mapped onto FPGAs. Indeed, the target chaos algorithms comprise code segments that can be unrolled into pipelines or partly executed in parallel. Although FPGAs are becoming attractive in real-time applications, investigations outside real-time applications may be less sensitive to power consumption. This enables FPGAs to be used in practical implementations in addition to traditional testing, verification, and validation. In engineering applications, chaotic systems can be employed in areas such as security. To that end, the reconfigurability of FPGAs; which enable algorithm upload and modification, and architectural modifications [31], is yet another attraction for targeting them when implementing chaotic systems.

One of the most important benchmarks for evaluating the performance of the discretization process is the Fmax that can be achieved by the target hardware. Fmax is expected to be much higher, using the digital circuitry, compared to its analogue counterparts. A combination of high Fmax and high accuracy is always desirable for real-time applications; however, this should also be correlated to the Throughput results of the digital FPGA-based implementation. In the presented cores, the highest Fmax was found to be 1329.79 MHz, which produced the highest Throughput in the non-scaled Euler-based algorithm with an accuracy of 16-bit float; this is a logical result as it corresponds to the implementation that requires the minimum resources. It is important to note that the reported Fmax in our work is the theoretical Fmax that the designed circuitry can attain independent of the device’s frequency limitations. However, the actual Fmax value is usually constrained by the speed of the slowest interface or clock networks in the utilized FPGA device, which is 800 MHz in our case [32]. The second best Fmax was found to be 988.14 MHz, corresponding to the RK-6 algorithm, emphasizing a dramatic improvement in robustifying the discretization algorithm, while achieving 74.3% of the highest possible Fmax. Comparing Fig 13 to both Figs 14 and 15, a perfect correlation is noticed, highlighting the consistency of the obtained results. The overall accuracy of the implementation depends on both the number of bits and the complexity of the discretization algorithm.

It is widely recognized that the employment of a smaller integration step size can significantly improve the precision of the discretization technique. As such, the reported results offer a high degree of flexibility in deciding on both the number of bits and the structure of the algorithm. Choosing RK-4, with a 32-bit float, offers a high-frequency operation of 555.86 MHz, even when using the scaled version of the Lorenz system that requires additional overhead to satisfy the required mathematical constraints on the values of the outputs. Traditional applications from the literature, with the common use of ADCs, usually target precisions of less than 16 bits. In our proposed implementations with precisions of 8 and 16 bits, changing the algorithm shows different patterns for increasing or decreasing Fmax; indicating a very high dependence on the physical utilization of the FPGA resources and how they are optimized.

When examining the effect of choosing either the scaled or the non-scaled version of the Lorenz system, it is clear that it follows the same argument, while exhibiting a strong correlation with the Throughput indicators in Figs 14 and 15. Developing aggregated performance indices that accurately assess trade-offs for each implementation is highly desirable. This approach, aligning with their relative importance, would significantly enhance the research reported in this paper [33–35].

As explained in the previous section, the Throughput, presented in Figs 14 and 15, was strongly correlated with Fmax. Increasing the accuracy, via increasing the number of bits, didn’t much impact the Throughput values. Limiting the evaluation to only the 8-bit float and the 16-bit float cases, as some of the cores of other precisions failed to synthesize due to the physical limitations of the used FPGA, it is clear that a pattern does exist for all different implementations. The absence of pattern applies to both the discretization algorithm and whether the scaled or the non-scaled structure of the Lorenz system was used. The best Throughput was achieved for the 16-bit float non-scaled Lorenz system, showing 265.96 Mpt/s and 12.77 Gbps. Increasing the accuracy, via adopting a more rigorous discretization algorithm, showed a consistent degradation in the overall Throughput. For the scaled Lorenz system, the Throughput ranged from [32.72, 69.06] Mpt/s and [0.86, 3.31] Gbps, while for the non-scaled version, the indicators changed to correspond to [25.99, 265.96] Mpt/s and [1.1, 12.77] Gbps.

Different appealing hardware area characteristics are attained for the developed Lorenz cores. The most economically occupied area, in LUTs, that produced the highest throughput is the non-scaled Euler algorithm with an accuracy of 16-bit float; the achieved area is 8092 LUTs. In addition, the number of DSP blocks and LRs exhibited less variation among implementations within [0, 810] for DSP Blocks and [7, 16] LRs. As per the target accuracies, implementations with higher accuracy consistently occupied larger areas. Common application areas, such as CBSC, that commonly require less than a 16-bit accuracy in modern systems due to ADC, can benefit from economical area utilization as achieved by Euler and RK-4 algorithms, with areas around 8000+ and 40000+ LUTs. However, higher accuracies can also benefit from the developed cores with areas that can fit mainly high-end FPGA systems (see Figs 10 and 11). Long-standing low-end FPGAs, such as Cyclone III (2007) with its different device models, are still recommended by their manufacturer [36]. Cyclone III FPGAs are produced with capacities that range between 5,136 and 198,464 LEs—each of a single LUT. Cyclone III can accommodate most of the developed Lorenz cores for different orders and accuracies. In all, Most economical in Combinational LUTs: scaled implementations are consistently larger than their non-scaled counterpart with an average increase of 11% ±6.91. Moreover, the best performance vector is holistically achieved by the Euler algorithm for the non-scaled version at an accuracy of 16 bits.

The total power consumption analysis presented in Fig 12 reflects that within the order of the discretization algorithm, the total power consumption increases with the increase in accuracy. Here, no outliers are found. The different cores, corresponding to the different discretization algorithms, consumed different total power but with comparable values that fall in the range [884.2, 1037.1] mW. As for the performance indicators vector that attained the highest Throughput, the least power consumption was attained by the Euler algorithm, 16-bit float, non-scaled, at 896.36 mW. Non-scaled RK-4 algorithm, 32-bit float, and RK-6 algorithm, 16-bit float, attained comparable power consumption of around 997 mW.

### 5.3 Closely-related work

**Fig 17:**
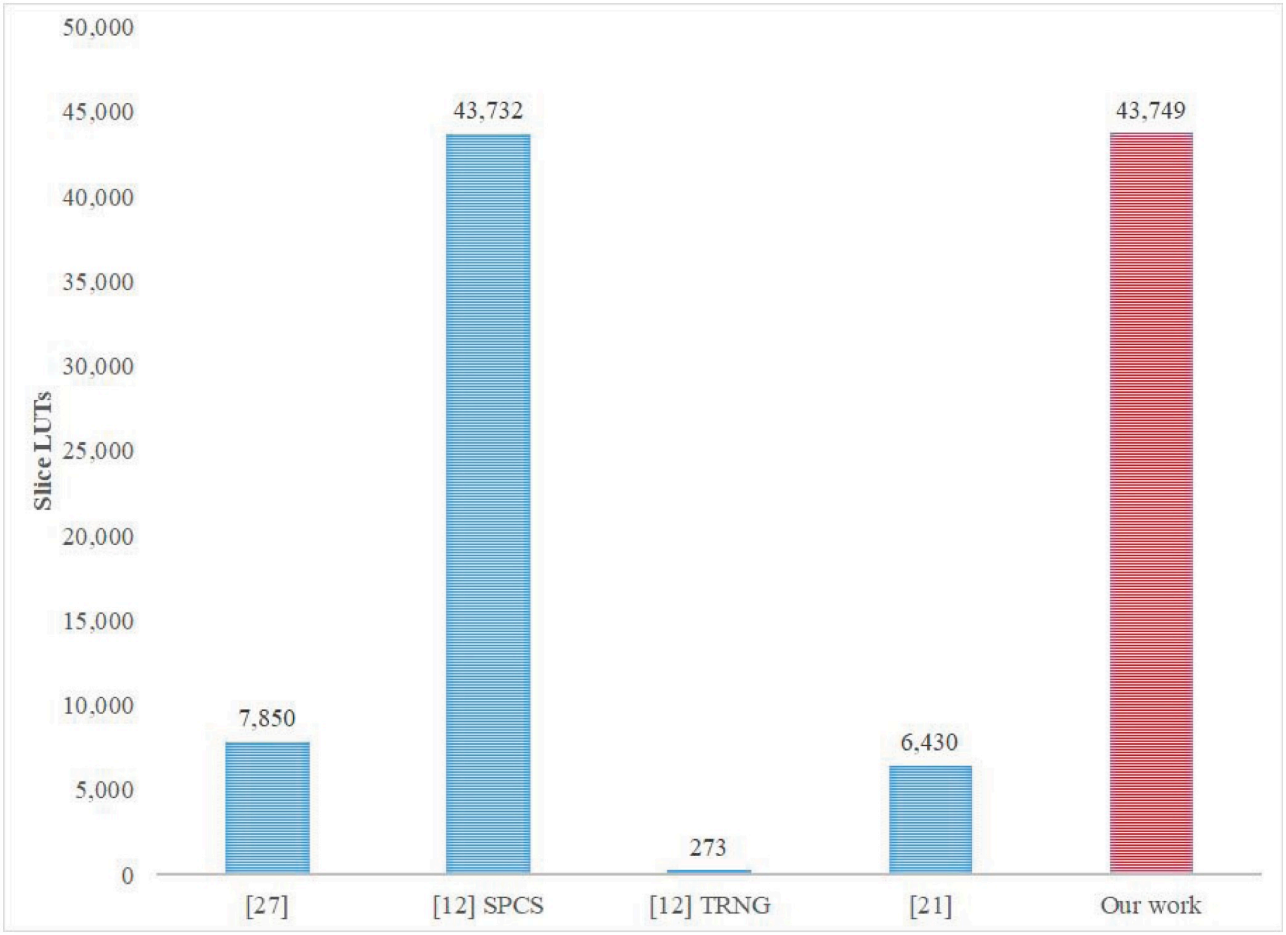
Number of utilized LUT Slices in different implementations [25, 26, 28] using a 32-bit RK-4 discretization algorithm.

**Fig 18:**
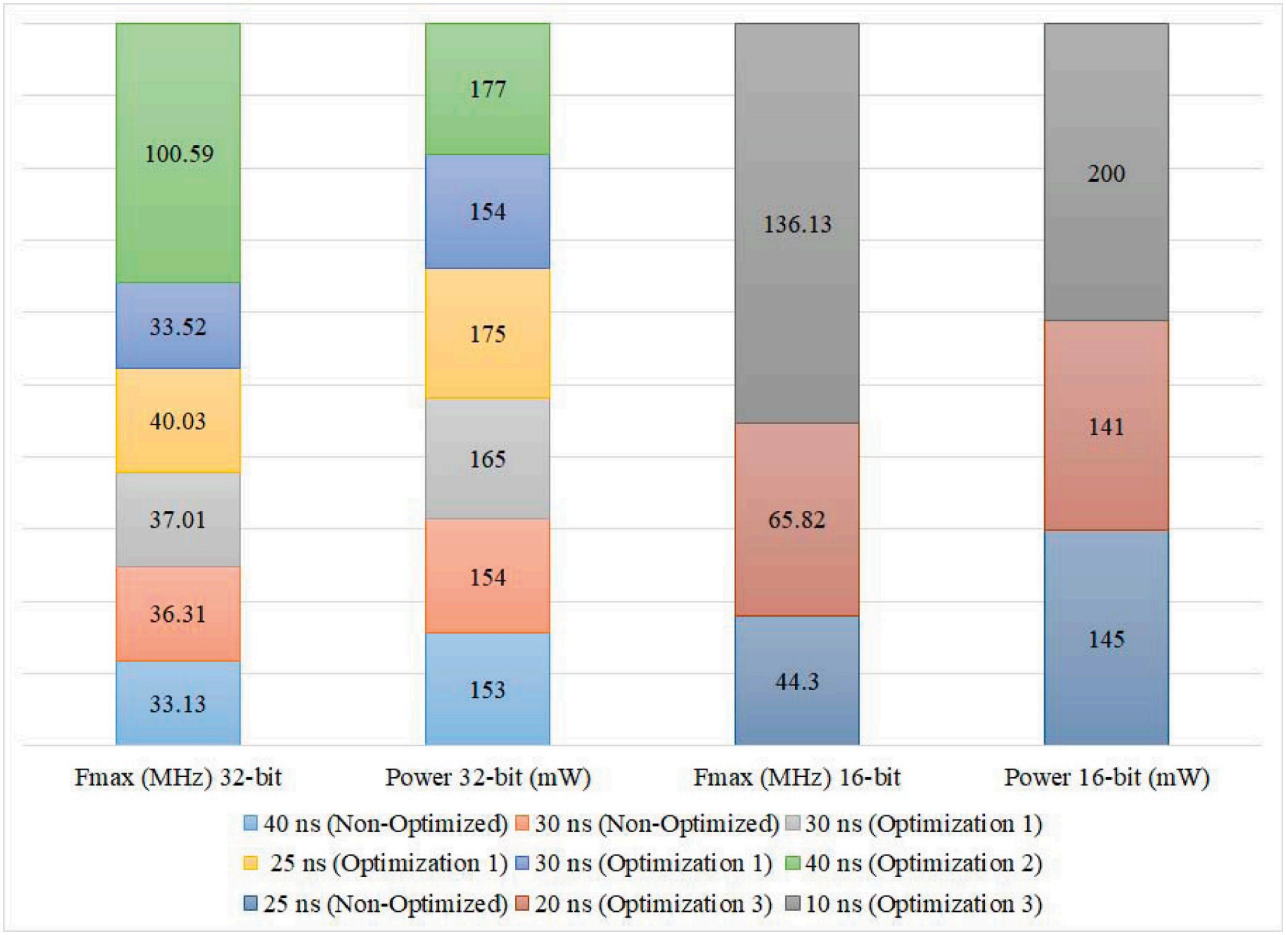
Power consumption and Fmax results of different 16-bit and 32-bit implementations [38] of Euler discretization algorithm.

In comparison with closely-related work [25, 26, 28, 37, 38], most reported investigations targeted RK-4 Lorenz discretization with an accuracy of 32 bits. Similar work comprises the development of FPGA implementations for wireless hyperchaotic communication systems [37], 3D chaotic systems [25, 28], chaotic generators [38], and chaotic TRNG [26]. Table 1 presents the reported Fmax in [25, 26, 28, 37]. The best-reported frequency is 390.067 MHz [25], which is somewhat lower than the frequency achieved in our proposed cores (see Fig 13). The best-reported throughput was 80 Mbps [37] and 159 Mbps [26] as compared with our 5.34 Gbps achieved with the same specifications; the attained speedup is 33.6 times the throughput reported in [26]. Fig 17 presents the number of utilized LUT slices achieved in different implementations [25, 26, 28] using a 32-bit RK-4 discretization algorithm. The results show a comparable area size (43,732 LUT Slices, Virtex VI, [25]) with our corresponding implementation (43,749 LUTs), while targeting an SPCS application. However, more economical area sizes were reported in [25] (7,850 LUT Slices, Virtex VI), [28] (6,430 LUT Slices, Cyclone IV), and [26] (273 LUT Slices, Virtex VI) within a TRNG application. As for power consumption, 16-bit and 32-bit implementations [38] of the Euler discretization algorithm are shown in Fig 18. The reported results show the attainment of lower power consumption (150–200 mW) than those presented in Fig 12 (895–910 mW) for the same discretization algorithm and accuracy but at significantly lower Fmax (see Fig 13).

**Table 1:** Reported maximum frequency (Fmax) in [25, 26, 28, 37] and the result attained by the comparable implementation presented in this paper.

Ref	Device	Fmax (MHz)	Comparable Implementation (This paper; MHz)
[37]	Virtex II	25.364	558.04
	Virtex V	36.271	
	Virtex VI	28.507	
	Virtex VII	35.842	
[25]	Virtex VI	390.067	
[26]	Virtex VI	293.815	
[28]	Cyclone IV	104.58	

### 5.4 Limitations and future work

Some limitations are identified for the proposed investigation on the application and implementation levels. From the chaotic systems perspective, without loss of generality, the developed cores in this paper were focused on the discretization process, using different algorithms, integration steps, and data precisions. For applications in the field of secure communication, when using cryptography or other chaos-based shift keying techniques, it is very well known that most of the computational effort is done in the synchronization process between the transmitter and the receiver. A similar argument applies to other chaos-based applications, such as TRNG. Consequently, more investigations will be required to explore the expected overhead in the computational effort, when adding more lines of HDL code to the FPGAs or addressing the latency of expected networking operations. In addition, dealing with other structures of chaotic systems that involve non-autonomous structures and/or hyperchaotic multi-dimensions will surely add more complexity to the proposed analysis and design, proposed in this paper. However, we hope that the work presented in this paper sets an example and provides implementation patterns that would enable such future developments.

In terms of the implementation, the proposed cores are limited to the available logic area in the target FPGA, namely the Stratix IV. To that end, some high-order implementations were over-mapped and results were not possible to obtain, specifically for the RK-6 algorithm (see Figs 13 through 15). On the processing level, pipelining the proposed cores is possible and may lead to significant performance characteristics. Work in progress includes mapping the developed FPGA cores to the communication interfaces of the DE4 Board with its Stratix IV FPGA to enable communication applications. To this end, pipelining chaotic systems can benefit from the sequential nature of some real-time communication options.

A variety of improvement opportunities are identified for a set of promising lines of future research work. The work presented in this paper addressed the Euler discretization method, which is considered simple enough, but requires a relatively small integration intermediate step to ensure stability and accuracy, followed by the RK-4 method, which is the most widely used algorithm, as it is considered the best compromise between simplicity and accuracy. Both algorithms were almost successful for all data precisions and operating frequencies. For future work, it is suggested to try other discretization methods, such as the Heun algorithm which needs only two integration steps and could be a better upgrade for the Euler method. In addition, other higher-order methods, similar to the RK-6 could be explored in an attempt to avoid the scenarios where the implementation failed, given the constraint of the used hardware. This can include Bogacki-Shampine and Dormand-Prince algorithms. Moreover, the effect of the discretization methods on the accuracy of reconstructing transmitted messages in CBSCs and the integrity of the standards for TRNG are possible extensions to this paper. Comparing the effect of the calculations overhead, using discretization, against directly using discrete chaotic systems, such as the Logistic or the Hénon maps, would be an interesting exploration for future work as well.

In hardware, implementing chaotic systems for heterogeneous computing systems, such as Graphical Processing Units (GPUs), Digital Signal Processors (DSPs), and their partitioned combinations can promise appealing implementation and performance characteristics. Furthermore, investigating the real-time embedded systems aspects and intercommunication scenarios can lead to a better understanding of application details. Indeed, the available variety of performance analysis indicators utilized in the evaluation process can enable the development of classification frameworks that can rank implementations according to their effectiveness [33, 34, 39].

## 6 Conclusion

In this paper, the problem of implementing continuous-time chaotic systems, using reconfigurable digital hardware was investigated. Different implementations were explored while using three discretization algorithms that correspond to simple (Euler), high (RK-4), and very high accuracies (RK-6). A variety of precisions were attempted, ranging from 8 to 64 bits, while evaluating the maximum operating frequency that can be obtained. Correlation between the different implementations and their corresponding throughputs, power consumptions, and area utilization were analyzed for a given Stratix IV FPGA, while conducting a comprehensive comparison with similar work, reported in the literature. The advantages, limitations, and possible extensions to the work presented in this paper were stated while providing illustrative comparisons in the form of tables and charts. In addition, future work that targets adding relevant applications such as CBSCs and TRNG was suggested. The unique investigation of the RK-6 discretization algorithm was highlighted, using different scenarios, including the additional overhead computational effort to implement scaled-magnitude outputs, for the used chaotic Lorenz system. This significant contribution can pave the way for implementing highly accurate and fast real-time CBSCs, with encryption.

### Appendix

Table 2 presents the acronyms used throughout the manuscript and their definitions.

**Table 2:** List of acronyms.

Acronyms	Definition
ADC	Analog to DC Converter
ASIC	Application-Specific Integrated Circuit
CBSC	Chaos-Based Secure Communication
DSP	Digital Signal Processor
FPGA	Field Programmable Gate Array
FP	Floating Point
FSM	Finite State Machine
Fmax	Maximum Frequency
GPU	Graphical Processing Unit
HDL	Hardware Description Language
HW	Hardware
LUT	Lookup Table
LE	Logic Elements
LR	Logic Register
ODE	Ordinary Differential Equation
Op-Amp	Operational Amplifier
RK-4	Runge-Kutta 4
RK-6	Runge-Kutta 6
PMSM	Permanent Magnet Synchronous Machines
SR	Stiffness Ratio
SW	Software
TTL	Transistor-transistor Logic
TRNG	True Random Number Generation
